# Chih-chen Wang: the social responsibility of scientists

**DOI:** 10.1093/nsr/nwaa299

**Published:** 2021-01-19

**Authors:** Chao Gu (顾超)

**Affiliations:** Department of History of Science, Technology and Medicine, Peking University, China

## Abstract

Chih-chen Wang is a distinguished biochemist and molecular biologist, and an Academician of the Chinese Academy of Sciences (CAS). From 2008 to 2013, she was a vice chairperson of the National Committee of the Chinese People's Political Consultative Conference (CPPCC). As a young researcher, Wang participated in research on insulin. Since the 1990s, she has been focusing on the study of protein folding, and has initiated a new research area of isomerase and molecular chaperones in China.

In this interview, Chih-chen Wang elaborates on the social responsibility of scientists by drawing on both her scientific research and CPPCC experience. In Wang's view, what China really needs are intellectuals with independent thinking and strong social responsibility, who are able to provide the government valuable advice and communicate with the public to increase society's scientific literacy. She also hopes that female scientists can be more confident and gain greater attention and support from society.

## PROTEIN FOLDING RESEARCH STARTED FROM INSULIN

***NSR:*** Research seems to be a life-long calling for you. All these years you’ve spent day after day, morning until night in the laboratory. What has been the driving force for you?

***Wang:*** Like others of my generation, I as a kid received education from the newly founded People's Republic of China. Patriotism, loyalty and a willingness to work hard became a part of our DNA. After the Cultural Revolution, once experiments could go ahead, we didn’t want to waste a minute. At that time, we had to wait a week or two for a chance to use the fluorescence spectrophotometer. Once, in order to make it to my registered slot, I cycled for an hour and a half in strong wind and heavy rain to get to the lab on time. I was soaked to the bone. Studying hard and being conscientious were habits that formed during our childhood, which were related to the education of that time, that is, we had to be strict with ourselves.

***NSR:*** You’ve said before that your career started from China's insulin research program, how did that experience influence your later research?

***Wang:*** I joined the insulin biochemistry group during the Cultural Revolution. My job was to prepare various insulin derivatives through chemical modification, study their physical, chemical and biological properties in solution, and analyze the relationship between their structure and function. That was the real beginning of my scientific career and prepared me for my later research at the Deutsches Wollforschungsinstitut in Germany as an Alexander von Humboldt fellow. It also gave me a foundation in protein biochemistry that would be important to my future research on protein folding, which began at the National Laboratory of Biomacromolecules in the early 1990s. It was rather late, yet very fortunate for me.

***NSR:*** You once said that compared to the Nobel Prize-winning work of Christian Anfinsen of the United States, which was driven by fascination and free exploration, China's insulin synthesis was more task-oriented. It also achieved some important results and was on the verge of major breakthroughs but just failed to push open the window. Is this a common phenomenon in scientific research? [Christian Anfinsen shared the 1972 Nobel Prize in Chemistry with Stanford Moore and William Howard Stein for their work on the structure and function of ribonuclease.]

**Figure 1:**
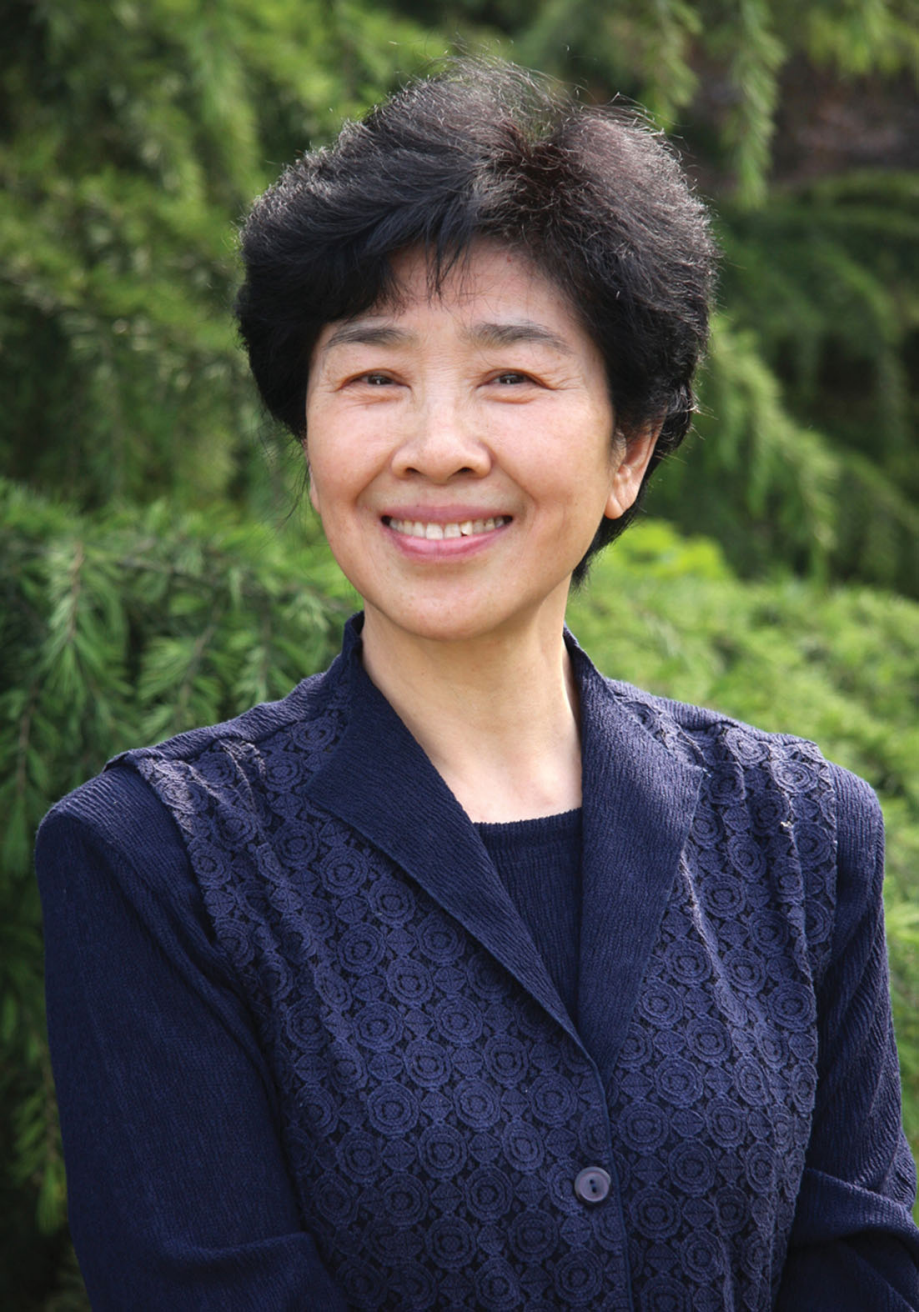
Prof. Chih-chen Wang is an expert in protein folding and was vice chairperson of the National Committee of the Chinese People's Political Consultative Conference from 2008 to 2013. *(Courtesy of Prof. Chih-chen Wang)*

***Wang:*** I can’t say it's a rule. Youyou Tu and her collaborators’ Nobel Prize-winning discovery of artemisinin was also task-oriented, which started from the investigation of traditional Chinese medicine, and in the end resulted in an effective new drug for malaria.

Throughout history, basic research is, in essence, small science done by small teams of people or even just one scientist. The free exploration of these small teams led to most of the major scientific breakthroughs and innovations.

On the other hand, big sciences have also been developed in modern society, such as the Manhattan Project, the Apollo Program and the Human Genome Project. The Large Hadron Collider (LHC) and the ITER nuclear fusion energy program currently going on in Europe are also examples of big science, bringing together many outstanding scientists from around the world. But big science programs are also based on the work of individual scientists, which, once successfully developed to a certain extent, is then integrated and refined by the program to obtain bigger achievements. In this regard, China is in a good position since we can take advantage of the merits of our ‘whole-nation system’.

Big science programs are also based on the work of individual scientists, which, once successfully developed to a certain extent, is then integrated and refined by the program to obtain bigger achievements.

—Chih-chen Wang

***NSR:*** What have been your main discoveries in protein folding?

***Wang:*** My work on protein folding issues raised in the total chemical synthesis of insulin. At the beginning of the program, Chen-lu Tsou's team successfully separated and recombined the A and B chains of natural insulin, thus clarifying the route of synthesis and successfully integrating the two chemically synthesized chains into the active form. But at that time, the mechanism of the folding and integration of the two chains was not fully understood.

It was not until the Reform and Opening-up in China began that Prof. Tsou was able to start exploring this problem at the Institute of Biophysics, CAS. I was in luck and got to participate in this research, and investigated the catalyzing function of protein disulfide isomerase (PDI).

Together with Prof. Tsou, I proposed the hypothesis that PDI acts as both an enzyme and a chaperone in promoting protein folding. Our hypothesis was contrary to the authoritative view at the time, but the paper was quickly cited by many scientists and was ranked as one of the 10 most cited papers in mainland China in 1997 and 1998.

After that, we further characterized these two activities of PDI, and concluded that it's only through the cooperation of the two activities of isomerase and chaperone that PDI is able to play the role of folding enzyme to catalyze the folding of peptide chains and the formation of disulfide bonds. This hypothesis is now well-accepted by international peers, and PDI’s function as a folding enzyme has been elucidated in an increasing number of physiological and pathological processes.

## ADVICE AND SUGGESTIONS FOR THE GOVERNMENT

***NSR:*** Many distinguished scientists withdraw from scientific research when they become science and technology officials or managers. But when you served as a vice-chairman of the CPPCC, in spite of it being a busy role, you kept going with your research. Why was that?

***Wang:*** I had never wanted to be a politician and was surprised to be elected for this job. But since it was entrusted to me, it was my responsibility to step up. I was unfamiliar with matters of politics so I chose to bring my tried and tested approach on research to this new role. I used the same prudence and diligence to study the official documents and to solicit advice from people around me. I was determined to improve myself through practice and to fulfill my duties of political deliberation, consultation and democratic supervision.

I was not a full-time politician, so I was still able to continue with research. During that period, I began to focus on training young scientists and encouraging them to be independent. Most scientists in my group have not been trained abroad, but they are well trained and some are now principal investigators.

***NSR:*** What kind of recommendations did you provide as CPPCC vice-chairperson?

***Wang:*** My recommendations mainly focused on the scientific research system and student cultivation. I once commented that the relationships between some tutors and their students were like the relationship between a shepherd and her sheep. To illustrate the problem, I once told an anecdote: *Prof. Z met a student in the elevator and asked, ‘Who is your supervisor?’ The student replied, ‘I am Prof. Z’s student.’* When I finished, everyone laughed. The professor had dozens of students under his name but did not know many of them, and some of his students did not know him either. Some Chinese professors were seeking fame and personal gain, but were irresponsible to research and their students. Unfortunately, this kind of situation is still found in Chinese academia.

Some scientists publish dozens of articles a year, but we all understand that it is impossible to be that quick to do the experiments, write the papers and revise each of them for many times. It is obvious that the works are not their own and they have not written these articles, and fraudulent results could be published. The result is, as we have seen in recent years, there have been several serious paper retraction events.

Now, an illegal academic paper production chain has formed in China. We have to crack down on this harmful industry. This phenomenon is also related to our evaluation system, especially in some disciplines, like medicine. Academic research publications are required for the promotion of clinical physicians, but for most physicians this is unnecessary and impractical. A physician in China who treats tens or even hundreds of patients a day is hardly going to have time for doing their own experiments and writing papers, but she/he can be a deeply experienced clinician and make an important contribution to the community. I once suggested to Prof. Yi Rao, president of Capital Medical University, that medical researchers and clinical physicians be independently evaluated, allowing both to be well paid and fully recognized.

Now, an illegal academic paper production chain has formed in China. We have to crack down on this harmful industry.

—Chih-chen Wang

***NSR:*** After stepping down from your role at CPPCC, did you continue to engage in this kind of work on science policy?

***Wang:*** After stepping down from CPPCC, I was elected to be president of the China Women's Association for Science and Technology (CWAST), which I took on until the end of 2019. The aim of CWAST is to strengthen the self-confidence of female scientists, technicians and students; encourage them to achieve; arrange for them to contribute their expertise to support poverty alleviation and benefit society; and engage in outreach to expand their social awareness and boost their sense of social responsibility.

We set up the ‘Service Award for Female Scientists’ to honor their contributions to social development. We also help them to solve real-life problems. For example, at our recommendation, the age limits of some scientific funding opportunities and awards, like the National Science Fund for Distinguished Young Scholars, for female scientists was extended by two or three years to compensate for their maternity leave and childcare. We also organized activities and events for female scientists, such as the ‘Walk or Jog for Health and Happiness’ activity and the annual ‘Science Meets Art’ concert co-organized with the Central Conservatory of Music.

Among all the Nobel laureates, less than 5% are female. The proportion is even lower in physics, chemistry and economics. In China, only about 5–10% of ‘top scientists’ are women. There are social prejudices here to a certain extent. I hope that the female scientists, accounting for about 40% of the total scientific workforce, will receive more attention and support from society.

***NSR:*** Why are there so few female university presidents and female scientific leaders? Are there problems of gender discrimination in China's scientific community?

***Wang:*** Gender equality is a basic state policy in China. Females of my age benefited a lot from this policy. Personally, I have not felt discriminated against as a female scientist. But in recent years, there seem to be more and more gender inequality problems emerging.

According to the surveys and discussions of the All-China Women's Federation and CAS, the female ratio of undergraduate and graduate students is basically 50% in China, and even higher in some majors such as life sciences and social sciences. Yet there are only 10–15% female professors, less than 10% female university presidents or chief scientists of large projects, and about 5% female CAS academicians.

There's something wrong related to our social ethos. Some young women believe that for women ‘doing well isn’t as important as marrying well’. Some want to enjoy a luxury lifestyle but don’t consider hard work a good way to succeed or to achieve. As one young woman put it, ‘I’d rather cry in a BMW than laugh on a bike’. In this regard, it is a failure of our media and social education. The media should tell more stories about science and scientists, especially young and female scientists, so that the younger generation can develop a healthier sense of self-awareness and self-expectation. The whole nation would benefit from the increased ability of the younger generation to innovate.

But instead, the media seem always to be asking female scientists ‘How do you balance your family and career?’. They never ask this question to male scientists. I totally agree with Prof. Nieng Yan [a female structural biologist] that this is an obvious prejudice. Males also have family responsibilities and they should also think about this question. It is a great pity that some very clever female students, when faced with marriage, family and children, may choose to lower their expectation of a career in science. I hope that more girls will refuse to give up on their ambitions, overcome the challenges they face and have the courage to keep going.

***NSR:*** How should we change these situations?

***Wang:*** There have been positive changes both in China and worldwide. The proportion of female XPLORER Prize [a non-governmental award for young Chinese scientists and technologists aged 45 and under] winners increased from 10% in 2019 to 12% in 2020. The increase was only 2%, but it's definitely a good start. Out of the eight 2020 Nobel Prize winners in natural sciences, three were women. This is a great encouragement to all female researchers around the world.

As for policy, I think we should stipulate a certain proportion of female leaders in education, science and technology. Women have the ability and should shoulder the responsibility of participating in high-level leadership. We should also promote the development of kindergartens, primary schools and middle schools, to free up female scientists from family affairs.

Women have the ability and should shoulder the responsibility of participating in high-level leadership.

—Chih-chen Wang

Most importantly, we need to correct the backward social ethos, so that girls can establish healthy values. They should empower themselves and be confident, independent and audacious. We should also guide all of society, especially males, to respect females. Everybody, no matter male or female, should cooperate with each other to fulfill their duties in work and life.

## THE SOCIAL RESPONSIBILITY OF SCIENTISTS

***NSR:*** Do you think that Chinese scientists can meet the scientific standards, proposed by R.K. Merton, of universality, commonality, selflessness and organized skepticism?

***Wang:*** Chinese scientists cannot be separated from society since they are not closed off in an ivory tower. Therefore, all kinds of thoughts and behaviors in society are bound to affect scientists. Some scientists are also founders of tech companies. If they mislead the public in order to promote their own products, this is not selfless. Standardized scientific ethics education is relatively lacking in China. In recent years, we have issued some policies and some universities are establishing scientific ethics courses. But I think it's also necessary to run such courses for middle school students. These basic ethical rules are more important than scientific knowledge and skills.

Organized skepticism is the basis of research. I once visited the Memorial of Comrade Chen Yun. There, I found a motto written by him, which reads ‘following neither authorities nor books, only facts; exchange, compare and repeat’. I personally identify with this motto. In my understanding, the first half elaborates on the essence of scientific spirit while the second half presents a down-to-earth interpretation of scientific methodology.

***NSR:*** In addition to following academic ethics, what do you think are the other social responsibilities of scientists?

***Wang:*** Some scientists say that we are ‘playing with science’ with the money of taxpayers. However, I think it's our responsibility to benefit society through our research. When selecting the direction for our research, including that of basic research, we should prioritize research which is scientifically significant and which can promote national security, the economy or public health. Actually, many major developments in human history came about due to great intellectuals who were willing to serve society.

Scientists must have a sense of social responsibility and a sense of mission. By ‘sense of social responsibility’, I mean the belief that a scientist should be responsible to the state and the people, and should try to do something for society beyond his or her own research, like popularizing science. A sense of mission may be something higher, such as giving up their existing living and working conditions and doing something more difficult for the good of our society. It is a higher realm—never being satisfied with existing achievements and comfortable conditions, and instead always being willing to give everything up and start from scratch to do bigger, tougher things for the society. China definitely needs intellectuals with insight, vision, ability, and a sense of responsibility and mission.

When I was the president of the Chinese Protein Society and CWAST, I required that after each academic meeting, the participants, including top scientists, give popular dissemination talks at local universities and middle schools.

***NSR:*** It's important for scholars of humanities and social sciences to contribute their independent ideas, which would lead to the progress of human society. However, for natural scientists, their scientific ideas are difficult for ordinary people and even other scientists to understand. So what impact does the independent thinking of scientists have on society?

***Wang:*** Be it natural science or social science, the essence of independent thinking is the same: to seek the facts following neither the superiors nor books, neither the mainstream nor the existing authority on something, but instead following the science and thinking only of the interests of the country and the people.

Suggestions from strategic scientists are extremely important for policy-making. For example, scientific problems are involved in many large-scale projects related to national security, economic development and the fundamental interests of the people. Scientists should selflessly offer their ideas on such social matters. At the same time, officials should encourage communication and debate among people with different ideas. Sometimes, ideas from a minority are significant to make the right decisions. Prof. Wanli Huang provided his opposing opinion on the construction of the large Yangtze River Dam, which was valuable and a good example of Chinese scientists’ independent thinking.

It's our responsibility to benefit society through our research.

—Chih-chen Wang

***NSR:*** Can Chinese scientists stick to their own scientific judgment on major public or social issues?

***Wang:*** Chinese scientists of an older generation are highly sensitive to major public or social issues and have a strong sense of responsibility. There are many examples of how they stuck to their independent scientific judgments and fought against pseudoscience.

From the 1980s to the 1990s, there was a big scam involving the claim that water can be transformed to oil. It was ‘authenticated’ by 10 university professors, encouraged by some officials and invested in by many companies. In 1995, Zuoxiu He, a CAS academician, published an article in *China Science Daily*, questioning the veracity of this ‘water to oil’ claim. After that, 41 CPPCC members from the science and technology sector, including Zuoxiu He, Zhongxian Zhao, Chen-lu Tsou and Wenjun Wu, published another article in *China Science Daily*, jointly calling for investigation of this ‘technology’ and its destructive consequences for the economy. The scientists invited the ‘inventors’ of this ‘water to oil’ technology to an open debate. The ‘inventors’ slunk away without facing the challenge.

There was also a ‘molecular biologist’ who boasted that he had invented ‘a traditional Chinese medicine with magical curative effect on cancer and coronary heart disease’. At its appraisal conference, attended by more than 50 experts, Prof. Chen-lu Tsou pointed out that the ‘molecular biologist’ had made basic mistakes in his enzymology experiment—mistakes so simple that anyone who had read a biochemistry textbook could identify. Astonishingly, some newspapers criticized Prof. Tsou as an ‘ignorant fake authority who never produces but always destroys’ who ‘should be penalized using Party discipline and state law’.

I could name a whole number of similar events, like ‘nucleic acid nutrition’, the ‘gene Queen’, the ‘holographic embryo theory’, the ‘cloning of 206 human tissues and organs within five years’, ‘Hanxin’ (a fake integrated circuit chip) and ‘Qiu's rodenticide’. In all these cases, Chinese scientists were brave enough to expose pseudoscience and safeguard the dignity of real science.

Younger Chinese scientists, who are active today, are also able to uphold independent scientific judgment and speak out publicly. There are two recent examples. One is that in 2016, a Chinese group published a top journal article claiming that they discovered a new gene editing technology, which was praised as ‘another CRISPR’ and a real breakthrough. But a group of scientists soon found that the experimental results could not be reproduced in a lot of labs. They discussed the issue publicly and the paper was finally retracted in 2017. The authors of the original article were young scientists and I hope that they can learn from the incident and make their works solid in the future.

Another example is the notorious CRISPR-baby event. Chinese scientists were among the first to respond. Prof. Guoqiang Bi and 122 other Chinese biomedical scientists issued a joint statement, resolutely opposing and strongly condemning this crazy violation of scientific ethics. They clearly showed the international community the attitude of Chinese scientists. We have the courage and integrity to fight against such rogue behaviors and safeguard scientific ethics.

In all these cases, Chinese scientists were brave enough to expose pseudoscience and safeguard the dignity of real science.

—Chih-chen Wang

***NSR:*** How do you think scientists can offer effective advice for policy-making?

***Wang:*** Most CPPCC members are very conscientious in preparing their proposals and have put forward many valuable suggestions. Our Jiusan Society [one of China's participating parties] put forward some good suggestions, such as protecting the sources of the Yangtze River, the Yellow River and the Lancang River. The government adopted this to good effect. In 2013, I wrote an article in *China Science Daily* calling for ‘no fireworks during Spring Festival to reduce PM_2.5_’. I also wrote to the mayor of Beijing about this issue. The mayor took it seriously and sent officials to come and talk to me. They took preventive measures against the risks of fireworks such as arranging a lot of physicians and fire engines on duty. And in many other cities, fireworks have been banned to reduce haze and accidents.

Scientists have also made many proposals on education, especially basic education. Currently, the cost of education is high. If you want to enter a good university, you have to pay for expensive extra-curricular classes. And test-driven education is not conducive to fostering students’ creativity. There are many problems to be solved.

***NSR:*** Besides the institutionalized channel of the CPPCC, are there other channels for scientists to offer advice?

***Wang:*** CAS has academician consulting projects, which is a good channel. Every year, each academic division organizes consultations on several topics. These are all urgent and important social topics. The National Science Foundation of China also has similar consulting projects.

## COMMUNICATING SCIENCE

***NSR:*** It seems that in some major public incidents relating to fake-science rumors, the voice of the scientific community is relatively weak.

***Wang:*** I have mentioned some examples of the rapid and positive voices of the scientific community. But it is true that most academic societies and institutes tend to be silent in controversial social incidents. They may be afraid that their statements will be misused by the media, and some are just unable to give an appropriate response when events are developing so quickly.

A more important problem is that even within the scientific community, there is not much debate over different opinions. This may have something to do with the Chinese culture, which tends to prioritize harmony in social relations and discourages people from sticking to a principle if it means having to debate with each other and rock the boat. This is contrary to the basic spirit of science. Unfortunately, changing this situation would be a slow and difficult process.

***NSR:*** How can we prevent an ‘infodemic’?

***Wang:*** I think the most important thing is to think for yourself, instead of blindly following whatever you see on the Internet. Equipped with basic scientific knowledge and principles, one should be able to identify obvious nonsense, like technology that turns water into oil or a drug that can cure all diseases. Aside from that, we should establish highly reputable platforms to set

Scientists need to improve their narrative and communication skills, and be more patient when communicating with the public.

—Chih-chen Wang

up anti-rumor columns, which would be easy for the public to use.

***NSR:*** Science communication involves both scientists and the public. Ideally, it should be bi-directional: scientists disseminate knowledge to the public, but also actively listen to and dialogue with the public, enabling the public to engage with science. It seems that in China, we do not see much of this kind of two-way communication.

***Wang:*** Yes. I think Dr. Wenhong Zhang [head of the Shanghai COVID-19 Treatment Specialist Group] has done a very good job with this. His communication with the public is scientific, realistic, humorous and vivid. The public can understand what he's saying, so they trust him and hope to hear more from him. That is especially precious in the context of an ‘infodemic’. I think there are several factors that make him so fearless: his strong sense of responsibility as a Communist Party member, his benevolence as a physician and his selflessness as someone with real integrity.

***NSR:*** From time to time, scientists are criticized on the Internet. What's your opinion of this?

***Wang:*** I think the best way to solve this problem is to promote communication between scientists and the public. Scientists need to improve their narrative and communication skills, and be more patient when communicating with the public. It is scientists’ social responsibility to educate the public and increase scientific literacy. Chinese scientists did a good job of communication during the COVID-19 pandemic. They will do better in the future.

***NSR:*** Can scientists lead the social ethos?

***Wang:*** Historically, Chinese scientists and intellectuals have led changes of social ethos several times. During the 4 May movement of 1919, we introduced democracy and science to China and our Jiusan Society stressed patriotism, democracy and science. These are all good traditions. In 1956, the Communist Party of China (CPC) Central Committee issued a call to ‘march toward science’, which set off an upsurge of scientific development all over the country. In 1978, Chi Xu's reportage of ‘Goldbach conjecture’ caused a great sensation, and then the National Science Conference was held to announce the arrival of the ‘spring of science’, marking the beginning of the rapid development of science and technology in China.

From these cases, we can see that scientists can lead the social ethos with the support of the government. Today, China has entered a new era. The development of science and technology in China is the result of many correct strategies of CPC, as well as the indelible contributions of all scientists. We should empower scientists, and make their voice and advice better heard by the government and the public.

## Notes

Chao Gu is a postdoctoral fellow in the Department of History of Science, Technology and Medicine, Peking University, China.

